# CRISPR interference of nucleotide biosynthesis improves production of a single‐domain antibody in *Escherichia coli*

**DOI:** 10.1002/bit.27536

**Published:** 2020-08-29

**Authors:** Jenny Landberg, Naia Risager Wright, Tune Wulff, Markus J. Herrgård, Alex Toftgaard Nielsen

**Affiliations:** ^1^ The Novo Nordisk Foundation Center for Biosustainability Technical University of Denmark Kongens Lyngby Denmark

**Keywords:** CRISPRi, *Escherichia coli*, growth decoupling, proteomics, single‐domain antibody

## Abstract

Growth decoupling can be used to optimize the production of biochemicals and proteins in cell factories. Inhibition of excess biomass formation allows for carbon to be utilized efficiently for product formation instead of growth, resulting in increased product yields and titers. Here, we used CRISPR interference to increase the production of a single‐domain antibody (sdAb) by inhibiting growth during production. First, we screened 21 sgRNA targets in the purine and pyrimidine biosynthesis pathways and found that the repression of 11 pathway genes led to the increased green fluorescent protein production and decreased growth. The sgRNA targets *pyrF, pyrG*, and *cmk* were selected and further used to improve the production of two versions of an expression‐optimized sdAb. Proteomics analysis of the sdAb‐producing *pyrF, pyrG*, and *cmk* growth decoupling strains showed significantly decreased RpoS levels and an increase of ribosome‐associated proteins, indicating that the growth decoupling strains do not enter stationary phase and maintain their capacity for protein synthesis upon growth inhibition. Finally, sdAb production was scaled up to shake‐flask fermentation where the product yield was improved 2.6‐fold compared to the control strain with no sgRNA target sequence. An sdAb content of 14.6% was reached in the best‐performing *pyrG* growth decoupling strain.

## Graphical abstract

Decoupling growth and production is a promising approach to improve cell factory performance. In this study, Landberg et al. inhibit the expression of selected genes in the nucleotide metabolism to stop cell growth and increase the production of a single‐domain antibody. Using proteomics, they show that the growth inhibited cells maintain their capacity for protein synthesis and do not enter stationary phase.

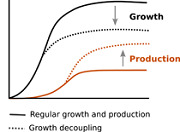

## 1 INTRODUCTION

Decoupling growth and production can significantly improve yield, titer, and productivity by dividing the bioproduction process into two phases; a growth phase where substrate is transformed into catalytic biomass, followed by a production phase where growth is stalled and substrate is utilized for product formation (Burg et al., 2016). Growth decoupling has been shown to increase the production of both biochemicals (Durante‐Rodríguez, De Lorenzo, & Nikel, 2018; S. Li, Jendresen, Grünberger et al., 2016; Willrodt, Hoschek, Bühler, Schmid, & Julsing, 2016) and proteins (Huber, Roth, Rahmen, & Büchs, 2011; Lemmerer et al., 2019), and a recently published strain design algorithm further highlights the possibilities of using growth decoupling to improve the production of a large number of small molecules in *Escherichia coli* (Venayak, von Kamp, Klamt, & Mahadevan, 2018).

Decoupling is generally achieved by natural or synthetic regulation of growth and/or induction of product expression. Unless necessary for the specific product, it is important that the decoupled cells do not enter the stationary phase to maintain cellular activity and production capacity. Natural regulation includes starvation for nutrients or other essential compounds (S. Li, Jendresen, & Nielsen, 2016; Matsuda et al., 2018; Willrodt et al., 2016), as well as regulation of environmental cues such as oxygen (Ge, Xu, Chen, & Zhang, 2015) or pH (Sun et al., 2014). Synthetic regulation usually involves synthetic circuits that interfere with growth and metabolic flux, and can be combined with sensing of environmental inputs. For example, temperature‐based decoupling can be achieved by coupling expression of a flux node to a temperature‐inducible promoter (Harder, Bettenbrock, & Klamt, 2018), or by using heat‐sensitive enzymes that shut down flux through a competing pathway upon temperature shift (Lynch, 2016).

Most commonly, synthetic regulation takes place on a translational or transcriptional level. However, posttranslational regulation of pathway proteins has also been shown to efficiently decouple growth and production. Tagging the first enzyme in a product‐forming pathway with a degradation tag that is cleaved off upon induction resulted in complete uncoupling of growth and production and rapid accumulation of high amounts of the biopolymer poly‐3‐hydroxybutyrate (Durante‐Rodríguez et al., 2018). In another study, inducible degradation of a glycolytic enzyme was used to redirect flux toward product formation and increase titer of *myo*‐inositol twofold (Brockman & Prather, 2015).

Translational regulation can be achieved through the use of orthogonal ribosomes for translation of the product or product pathway (Darlington, Kim, Jiménez, & Bates, 2018), or by stalling growth by inhibiting endogenous ribosomes (Mairhofer, Striedner, Grabherr, & Wilde, 2016). Combining inhibition of the native *E. coli* ribosomes with glycotransferase expression from T7 polymerase increased the glycotransferase product yield almost 12‐fold (Lemmerer et al., 2019).

Regulation of transcription has successfully been applied to inhibit cell accumulation and turn on production, often through synthetic genetic circuits (Lo, Chng, Teo, Cho, & Chang, 2016). By controlling isocitrate lyase expression with a degradable inducer, carbon flux could gradually be routed toward wax ester accumulation, improving wax ester yields almost fourfold during growth on acetate (Santala, Efimova, & Santala, 2018). CRISPR interference (CRISPRi) is an excellent tool for regulating gene expression on a transcriptional level (Larson et al., 2013). It can be induced to target gene(s) or cellular function(s) at a desired time point to increase precursor supply (Cress et al., 2017), redirect metabolic flux toward production and away from byproduct formation (Chang, Su, Qi, & Liang, 2016; Tian, Kang, Kang, & Lee, 2019), or to induce growth arrest by the inhibition of essential genes (S. Li, Jendresen, Grünberger et al., 2016; Shabestary et al., 2018). Partial CRISPRi‐based inhibition of citrate synthase GltA increased the productivity of butanol in the cyanobacteria *Synechocystis* (Shabestary et al., 2018), and CRISPRi‐based inhibition of *pyrF* improved the yield of mevalonate almost fivefold in *E. coli* (S. Li, Jendresen, Grünberger et al., 2016). To screen the *E. coli* genome for additional promising growth decoupling targets, we established a genome‐wide sgRNA library and screened it to identify several targets that increased the green fluorescent protein (GFP) production and inhibited growth (S. Li et al., 2020). The results from this study indicated an enrichment of promising targets among genes involved in biosynthesis of purines and pyrimidines.

Here, we construct CRISPRi‐based growth switches targeting the purine and pyrimidine biosynthesis pathway in *E. coli*, and screen for improved protein production. More than half of the screened growth switch targets display significant growth decoupling effects, with simultaneous growth inhibition and increase in GFP production. We apply the three top‐performing targets for production of two different expression‐optimized versions of a single‐domain antibody (sdAb or Nanobody®; Rennig et al., 2018). sdAbs are antibodies derived from camelids or cartilaginous fishes with potential use in various biotechnological applications, including as therapeutics (Wesolowski et al., 2009). Here, we see a significant increase in sdAb production for cultures with activated CRISPRi. To detect proteome‐wide changes induced by the expression of the CRISPRi system and inhibition of the specific targets, we conduct a proteomics analysis of all sdAb‐producing cultures. Proteomics shows that RNA polymerase sigma (RpoS) levels are significantly downregulated and ribosome‐associated proteins are significantly upregulated in the growth decoupling CRISPRi‐strains compared to the sdAb‐expressing control strains after 24 hr of growth. We conclude that although CRISPRi‐based repression of nucleotide biosynthesis stalls growth, it does not induce stationary phase response. Furthermore, the maintained ribosome content in the growth decoupled strains may explain the increase in sdAb accumulation. Finally, we apply the two top targets in shake flask fermentation and show that inhibition of *pyrG* increases sdAb yield 2.6‐fold compared to the control strain without sgRNA target, with sdAb levels reaching 14.6% of the total protein content.

## 2 MATERIALS AND METHODS

### 2.1 Media and materials

Lysogeny broth agar plates (LB, 10 g/L tryptone, 5 g/L yeast extract, 10 g/L NaCl) and 2xYT (16 g/L bactotryptone, 10 g/L yeast extract, 5 g/L NaCl) medium with appropriate antibiotics were used for cultivation and screening during cloning. Ampicillin, chloramphenicol, and kanamycin were used with working concentrations of 100, 50, and 50 μg/ml, respectively. Growth and production were carried out in M9 minimal medium with 0.1 mM CaCl_2_, 2.0 mM MgSO_4_, 1 × M9 salts, 1 × trace element solution, and 1 × vitamin solution, which was supplemented with glucose and appropriate antibiotics. The 10 × concentrated stock solution of M9 salts consisted of 6.8 g/L Na_2_HPO_4_ anhydrous, 3 g/L KH_2_PO_4_, 5 g/L NaCl, and 1 g/L NH_4_Cl, which had been dissolved in double‐distilled water and autoclaved. The 1,000 × concentrated stock solution of trace elements consisted of 15 g/L EDTA(Na_2_)·2H_2_O, 4.5 g/L ZnSO_4_·7H_2_O, 0.7 g/L MnCl_2_·4H_2_O, 0.3 g/L CoCl_2_·6H_2_O, 0.2 g/L CuSO_4_·2H_2_O, 0.4 g/L NaMoO_4_·2H_2_O, 4.5 g/L CaCl_2_·2H_2_O, 3 g/L FeSO_4_·H_2_O, 1 g/L H_3_BO_3_, and 0.1 g/L KI, which had been dissolved in double‐distilled water and sterile filtered. The 1,000 × concentrated stock solution of vitamins consisted of 10 mg/L pyridoxine HCl, 5 mg/L thiamine HCl, 5 mg/L riboflavin, 5 mg/L nicotinic acid, 5 mg/L calcium D‐pantothenate, 5 mg/L 4‐aminobenzoic acid, 5 mg/L lipoic acid, 2 mg/L biotin, 2 mg/L folic acid, and 0.1 mg/L vitamin B12, which had been dissolved in double‐distilled water and sterile filtered. Chemicals that were used in the study were purchased from Sigma‐Aldrich (Taufkirchen, Germany) and restriction enzymes and polymerase chain reaction (PCR) polymerases were purchased from Thermo Fisher Scientific (Waltham, MA). USER enzyme was purchased from BioNordika (Herlev, Denmark).

### 2.2 Plasmid and strain construction

The primers used in this study were ordered from Integrated DNA Technologies (Leuven, Belgium). All primers are listed in Table S1. Plasmid purification was carried out with the Macherey‐Nagel Plasmid Purification Kit (Dure, Germany) and cell transformation was carried out using the transformation and storage solution buffer method (Chung, Niemela, & Miller, 2006). *E. coli* DH5α was used for cloning and propagation. All strains and plasmids used in the study are listed in Table S2.

The sgRNA plasmids were constructed by Gibson assembly (Gibson et al., 2009). Primers containing a 20‐nucleotide target sgRNA sequence specific for each target gene were used to amplify pSLQ1236 (Larson et al., 2013). The linear fragment was then assembled according to standard Gibson assembly protocol. The sgRNA sequences were designed using CRISPy‐web (Blin, Ebdrup, Weber, & Lee, 2016) and are shown in bold in the primer list (Table S1). The psdAb‐TIR plasmids were constructed by USER cloning (Cavaleiro, Kim, Seppälä, Nielsen, & Nørholm, 2015; Nour‐Eldin, Hansen, Nørholm, Jensen, & Halkier, 2006). The translation‐optimized sdAb expression plasmids pET28a‐Nanobody®‐TIR^SynEvo1^ and pET28a‐Nanobody®‐TIR^SynEvo2^ and the pClodF13 origin of replication from pCDFDuet (Novagen) was amplified with Phusion U polymerase using primers jl130/131 and jl154/155, respectively. The PCR products were mixed with USER enzyme and incubated for 20 min at 37°C and 20 min at 25°C, followed by transformation to competent cells. The tetracycline‐inducible dCas9 was integrated into the attB‐186(O) site in the *E. coli* genome using the clonetegration method from St‐Pierre et al. (2013). The selection marker was excised using FLP recombinase and integration was confirmed using colony PCR.

### 2.3 Screening of sgRNA targets

Precultures were prepared by inoculation of biological triplicates of each sgRNA‐target strain in a 96‐deep well plate (96‐DWP) with 800 μl M9 medium supplemented with 0.5% glucose and 0.02% yeast extract (YE), and were grown overnight at 37°C, 250 rpm. The overnight cultures were inoculated with a 1:100 inoculum ratio (start optical density [OD] of ∼0.03) in two duplicate 96‐DWPs with 800 μl M9 medium supplemented with 0.5% glucose, and were grown at 37°C, 250 rpm for 24 hr. After 1 hr of growth, 200 ng/ml of anhydrotetracycline (aTc) was added to one of the duplicate 96‐DWP to induce the CRISPRi system. OD and fluorescence was measured after 12 and 24 hr of growth. OD was measured at 600 nm. For flow cytometry, samples were diluted appropriately and analyzed with an LSRFortessa (Becton Dickinson, San Jose, CA). The expression of GFP was detected using a 488‐nm long‐pass and a 530/30‐nm band‐pass filter setting. The forward‐scatter and side‐scatter was detected as small‐ and large‐angle scatters of the 488 nm laser, respectively. The results were analyzed with the FlowJo (Becton, Dickinson & Company, Franklin Lakes, NJ).

### 2.4 Single‐domain antibody production

Precultures were prepared by inoculation of biological triplicates of cells transformed with the sdAb and the sgRNA plasmids. Precultures were grown overnight at 37°C, 250 rpm in 24‐DWP in 2.5 ml of M9 medium with 0.5% glucose and 0.02% YE. For the small‐scale sdAb production experiment, overnight cultures were inoculated in duplicates to an OD of 0.03 in 24‐DWPs with 2.5 ml M9 medium with 1% glucose. For sdAb production in shake flasks, overnight cultures were inoculated in duplicates to an OD of 0.03 in 250‐ml shake flasks with 50 ml M9 medium with 1% glucose. The CRISPRi system was induced in half of the cultures after 1 hr using 200 ng/ml aTc. sdAb production was induced with 1 mM isopropyl β‐D‐1‐thiogalactopyranoside (IPTG) at OD 0.4. After 24 hr, 1 OD unit of culture was harvested and submitted for proteomics analysis. All samples from the deep well plate and the shake flask fermentation experiments, respectively, were run in the same proteomics analysis round, where label‐free quantification (LFQ) values of the measurable proteins present in the cell were determined. The sdAb content (%) for each strain was calculated by dividing the LFQ value of the sdAb with the total LFQ value.

### 2.5 Sample preparation for proteomics analysis

Frozen cells were kept at −80°C until processing of samples. Thawing of the cells was done on ice and any remaining supernatant was removed after centrifugation at 15,000*g* for 10 min. While kept on ice, two 3‐mm zirconium oxide beads (Glen Mills, Clifton, NJ) were added to the samples. Immediately after moving the samples away from ice, 100 μl of 95°C guanidinium HCl (6 M guanidinium hydrochloride, 5 mM tris(2‐carboxyethyl)phosphine, 10 mM chloroacetamide, 100 mM Tris–HCl pH 8.5) was added to the samples. Cells were disrupted in a Mixer Mill (MM 400 Retsch; Haan, Germany) set at 25 Hz for 5 min at room temperature, followed by 10 min in thermo mixer at 95° at 2,000 rpm. Any remaining cell debris was removed by centrifugation at 15,000*g* for 10 min, after which 50 μl of supernatant was collected and diluted with 50 μl of 50 mM ammonium bicarbonate. Based on protein concentration measurements, 100 μg protein was used for tryptic digestion. Tryptic digestion was carried out at constant shaking (400 rpm) for 8 hr, after which 10 μl of 10% trifluoroacetic acid was added and samples were ready for StageTipping using C18 as resin (Empore, 3M).

For analysis of the samples, a CapLC system (Thermo Fisher Scientific) coupled to an Orbitrap Q‐exactive HF‐X mass spectrometer (Thermo Fisher Scientific) was used. First, samples were captured at a flow of 10 μl/min on a precolumn (µ‐precolumn C18 PepMap 100, 5 µm, 100 Å) and then at a flow of 1.2 µl/min the peptides were separated on a 15 cm C18 easy spray column (PepMap RSLC C18 2 µm, 100 Å, 150 µm × 15 cm). The applied gradient went from 4% acetonitrile in water to 76% over a total of 60 min. While spraying the samples into the mass spectrometer, the instrument operated in data‐dependent mode using the following settings: MS‐level scans were performed with Orbitrap resolution set to 60,000; AGC Target 3.0e6; maximum injection time 50 ms; intensity threshold 5.0e3; dynamic exclusion 25 s. Data‐dependent MS2 selection was performed in Top 20 Speed mode with HCD collision energy set to 28% (AGC target 1.0e4, maximum injection time 22 ms, Isolation window 1.2 m/z).

### 2.6 Proteomics data analysis

For analysis of the thermo rawfiles, Proteome discoverer 2.3 was used with the following settings: fixed modifications: Carbamidomethyl (C) and variable modifications: oxidation of methionine residues. First search mass tolerance 20 ppm and a MS/MS tolerance of 20 ppm. Trypsin as enzyme and allowing one missed cleavage. FDR was set at 0.1%. The match between runs window was set to 0.7 min. Quantification was only based on unique peptides and normalization between samples was based on total peptide amount. For the searches, a protein database consisting of the reference *E. coli* proteome UP000000625 and the sequences of the sdAb (Rennig et al., 2018) and dCas9 (Larson et al., 2013) were used.

### 2.7 Computational analysis and visualization of proteomics data

For further processing and data analysis of the proteome dataset, only proteins with measurements in all samples were used (1,739 proteins for the DWP experiment). Differential expression analysis was performed using the EdgeR package (Robinson, McCarthy, & Smyth, 2009). Gene Ontology (GO) terms (Ashburner et al., 2000; The Gene Ontology Consortium, 2019) were obtained from current.geneontology.org/annotations/ecocyc.gaf.gz on 2 September, 2019, and GO analysis was performed by means of the *Piano* package using the method *Stouffer* (Väremo, Nielsen, & Nookaew, 2013). *p* values were adjusted for multiple testing using the Benjamini/Hochberg approach.

## 3 RESULTS AND DISCUSSION

### 3.1 Construction and screening of growth decoupling strains targeting purine and pyrimidine biosynthesis

**Figure 1:**
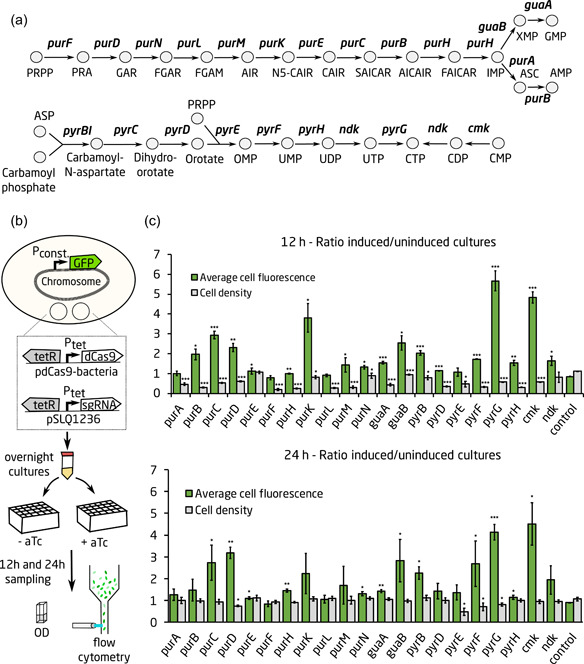
Screening the purine and pyrimidine biosynthesis pathways for growth decoupling targets. (a) The sgRNA target genes in the purine and pyrimidine biosynthesis pathways of *Escherichia coli*. (b) Experimental overview. (c) Ratio of OD and of GFP fluorescence in CRISPRi‐induced and uninduced cultures for each of the screened sgRNA targets after 12 and 24 hr of growth. OD and fluorescence were calculated as the average of three biological replicates. Standard deviations between replicates are shown as error bars. A two‐tailed *t* test was used to check for significant difference between the growth decoupling strains and the control; *p < .05; **p < .001; ***p < .0001. AICAR, 5′‐phosphoribosyl‐1′‐N‐(5‐amino)imidazole‐4‐N‐carboxamide; AIR, 5′‐phosphoribosyl‐1′‐N‐(5‐amino)imidazole; AMP, adenosine 5′‐monophosphate; ASC, adenylosuccinate; CDP, cytidine 5′‐diphosphate; CMP, cytidine 5′‐monophosphate; CRISPRi, CRISPR interference; CTP, cytidine 5′‐triphosphate; FAICAR, 5′‐phosphoribosyl‐1′‐N‐(5‐formylamino)imidazole‐4‐N‐carboxamide; FGAR, 5‐phosphoribosyl‐1‐N‐formylglycinamide, FGAM, 5‐phosphoribosyl‐1‐N‐formylglycinamidine; GAR, 5‐phosphoribosyl‐1‐N‐glycinamide; GMP, guanosine 5′‐monophosphate; IMP, inosine 5′‐monophosphate; N5‐CAIR; 5′‐phosphoribosyl‐1′‐N‐(5‐amino)imidazole‐5‐N‐carboxylate; OD, optical density; OMP; orotidine 5′‐monophosphate; PRA, 5‐phosphoribosyl‐1‐amine; PRPP, phosphoribosyl diphosphate; SAICAR, 5′‐phosphoribosyl‐1′‐N‐(5‐amino)imidazole‐4‐N‐succinocarboxamide; sdAb, single‐domain antibody; UDP, uridine 5′‐diphosphate; UMP, uridine 5′‐monophosphate; UTP, uridine 5′‐triphosphate; XMP, xanthosine 5′‐monophosphate [Color figure can be viewed at wileyonlinelibrary.com]

A total of 22 different genes in the nucleotide biosynthesis pathway were selected as targets to investigate the potential of using purine and pyrimidine biosynthesis genes as CRISPRi‐based growth switches (Figure 1a). The chosen targets are part of de novo purine biosynthesis (*purA, purB, purC, purD, purE, purF, purH, purK, purL, purM, purN, guaA, guaB*), de novo pyrimidine biosynthesis (*pyrB, pyrC, pyrD, pyrE, pyrF, pyrG, pyrH, ndk*), or the pyrimidine salvage pathway (*cmk*; Martinussen, Willemoës, & Kilstrup, 2011). sgRNAs targeting the different genes were cloned onto plasmid pSLQ1236 using Gibson cloning, resulting in 21 plasmids (we were not successful at obtaining a cloning construct for the sgRNA targeting *pyrC*). Each sgRNA plasmid was transformed together with pdCas9 into strain MG1655‐gfp, harboring a genome‐integrated GFP under constitutive promoter J23100 inserted 9‐bp downstream of *glmS* (Bonde et al., 2016). An empty sgRNA plasmid with no insert sequence as well as a wild‐type *E. coli* strain were used as controls. To compare growth and production of samples with the CRISPRi system induced or uninduced, overnight precultures were split in two and one was induced with aTc after 1 hr of growth. Samples for measuring growth and fluorescence were taken after 12 and 24 hr (Figure 1b).

Results showed that after 12 hr, 17 out of 21 induced targets displayed increased the GFP production compared to the respective uninduced control, with a fold change between 1.1‐ and 5.7‐fold (Figure 1c, upper plot and Figure S1a). Background fluorescence from the wild‐type control was negligible (data not shown). Inhibition of *pyrG* and *cmk* resulted in the highest GFP production levels. Growth inhibition could be seen in 19 out of the 21 targets, and 15 targets displayed simultaneous inhibition of growth and increase in GFP production (Figures 1c and S1b). After 24 hr, 12 out of 21 targets still showed a significant increase in production, with the fold‐change decreasing slightly to a range between 1.1‐ and 4.5‐fold (Figure 1c, lower plot and Figure S1c). CRISPRi‐based repression of *pyrG* and *cmk* still resulted in the highest production. In most cultures, the fluorescence had decreased compared to the 12 hr time point. Only four out of 21 CRISPRi‐induced strains had a lower OD compared to the respective uninduced control (Figure S1d); however, this can to large extent be explained by the decrease in OD that uninduced strains displayed between the 12 and 24 hr sample points. This decrease could also be seen for the wild‐type control strain (data not shown).

Cell size could have a significant impact on protein accumulation, as larger cells can contain higher amounts of protein. However, flow cytometry data of the CRISPRi‐induced strains showed that of all the screening targets, only *purK* had an (around twofold) increase in cells size compared to the respective uninduced control.

Overall, the growth switch targets *pyrG* and *cmk* were the best‐performing targets in the screen (Figures 1c and S1e). They were selected for further testing together with *pyrF*, which has previously been shown to work as an efficient growth switch for both protein and biochemical production (S. Li, Jendresen, Grünberger et al., 2016). Flow cytometry analysis of these strains revealed that the CRISPRi‐induced *pyrF* and *cmk* populations had a unimodal fluorescence distribution after 12 hr, with *pyrF* showing signs of a slight shift toward bimodality after 24 hr (Figure S1e). On the other hand, the *pyrG* population had a bimodal fluorescence distribution at both 12 and 24 hr, where part of the population produced GFP in similar levels as the control, and part of the population produced very high amounts of GFP (Figure S1e). This indicates that the *pyrG* strain is divided into two populations after CRISPR induction, where one consists of a growth‐stalled, high‐producing cells and the other consists of regularly growing and producing cells.

It is also worth noting that the strain used in our study, MG1655, has a mutation in *rph1* that interferes with expression of *pyrE*, which is located downstream or *rph1* (Jensen, 1993). Therefore, MG1655 is under pyrimidine limitation at higher growth rates (Jensen, 1993), which could potentially strengthen the growth inhibition efficiency of CRISPRi when targeting pyrimidine biosynthesis.

### 3.2 Improving sdab production using growth decoupling

**Figure 2:**
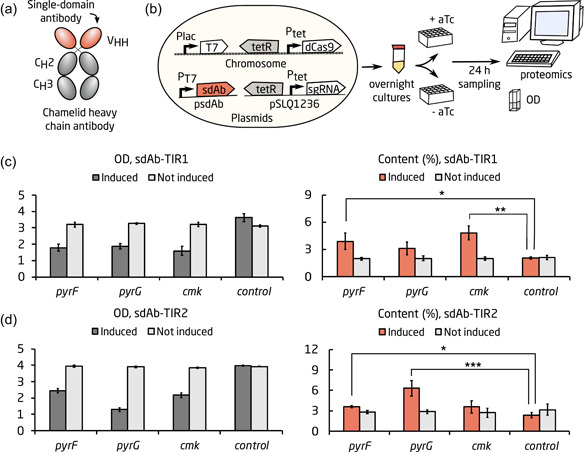
sdAb production in deep well plate. Application of the top‐performing growth switches *pyrF, pyrG*, and *cmk* for production of two expression‐optimized sdAbs with different translation initiation regions (TIR). (a) sdAbs are derived from the heavy chain of an antibody. (b) Experimental overview. (c) Growth and sdAb production after 24 hr for strains harboring psdAb‐TIR1 and sgRNA plasmids targeting *pyrF, pyrG*, and *cmk*. (d) Growth and sdAb production after 24 hr for strains harboring psdAb‐TIR2 and sgRNA plasmids targeting *pyrF, pyrG*, and *cmk*. For (c) and (d), the first bar graph shows OD and the second bar graph shows percent sdAb content. Cultures, where the CRISPRi system was induced, are shown in the dark gray (OD) or red (sdAb content). Uninduced cultures are shown in bright gray. The values were calculated as an average of three biological replicates. Error bars represent standard deviation of the replicates. A two‐tailed *t* test was used to check for significant difference between the strains; *p < .05; **p < .001; ***p < .0001. CRISPRi, CRISPR interference; OD, optical density; sdAb, single‐domain antibody [Color figure can be viewed at wileyonlinelibrary.com]

Next, we applied the *pyrF, pyrG*, and *cmk* targets for improving the production of a commercially relevant protein. sdAbs or Nanobody® (Figure 2a) are derived from immunoglobulin‐γ antibodies found in camelids (Hamer‐Casterman Atarchouch et al., 1993). They possess various interesting features compared to the commonly used monoclonal antibodies, such as smaller size, higher solubility, and increased stability (Wesolowski et al., 2009). sdAbs are commonly produced in *E. coli* as they generally do not require posttranslational modifications (Fernandes et al., 2017). They can be used as they are or fused to chemicals or protein domains, and have a great potential for applications within research, diagnostics, and as therapeutics (Wesolowski et al., 2009). The first sdAb on the therapeutics market was recently approved for treatment of a blood disorder (Chanier & Chames, 2019). We selected an sdAb for which the expression had previously been optimized in a study by Rennig et al. (2018). They developed two different translation‐optimized versions of the sdAb (pET28a‐Nanobody®‐TIR^SynEvo1^ and pET28a‐Nanobody®‐TIR^SynEvo2^). Both harbored changes in the six nucleotides upstream of the start codon, which significantly improved expression compared to the original construct (Rennig et al., 2018). To facilitate culturing, the tetR‐pTet‐dCas9 cassette was integrated into the phage 186 integration site in the genome of MG1655‐DE3 (Mundhada, Schneider, Christensen, & Nielsen, 2016) using pOSIP (St‐Pierre et al., 2013), resulting in strain MG1655‐DE3‐dCas9. To avoid plasmid incompatibility between the sdAb and sgRNA plasmids, the origin of replication for pET28a‐Nanobody®‐TIR^SynEvo1^ and pET28a‐Nanobody®‐TIR^SynEvo2^ were changed to ClodF13, resulting in psdAb‐TIR1 and psdAb‐TIR2, respectively. MG1655‐DE3‐dCas9 was transformed with psdAb‐TIR1 or psdAb‐TIR2 and sgRNA plasmids with targets *pyrF, pyrG*, and *cmk*. An sgRNA vector without targeting sequence was used a control. Precultures were grown in a 24‐DWP with 2.5 ml media overnight. The precultures were inoculated in duplicates into two 24‐DWPs with 2.5 ml fresh media. One of these was induced with aTc after 1 hr of growth. sdAb production was induced in all cultures at an OD of 0.4 using 1 mM IPTG. Samples were collected for OD and proteomics after 24 hr (Figure 2b). The proteomics data for the deep well plate experiment can be found in File S1.

**Figure 3:**
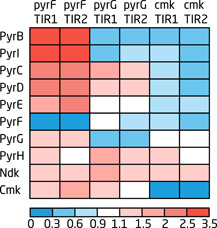
Heatmap of the fold‐change of protein levels in the pyrimidine biosynthesis pathway of the CRISPRi‐induced *pyrF, pyrG*, and *cmk* growth decoupling strains expressing psdAb‐TIR1 and psdAb‐TIR2. Fold‐change for the growth decoupling strains was calculated by dividing the CRISPRi‐induced *pyrF, pyrG*, and *cmk* strains with the respective (TIR1 or TIR2) CRISPRi‐induced control strain harboring the sgRNA control plasmid. The data can be found in Table S3. CRISPRi, CRISPR interference [Color figure can be viewed at wileyonlinelibrary.com]

The CRISPRi‐induced cultures showed significant growth inhibition, as the OD reached around half the OD of the uninduced controls (Figure 2c,d, Panel 1). The uninduced cultures harboring sgRNA targets all grew to a similar OD as the control strains. Upon induction of the respective target sgRNA, protein levels of PyrF, PyrG, and Cmk were decreased to 6%, 35%, and 10% respectively, compared to the uninduced control strain in the sdAb‐TIR1 strains (Figures 3 and S2a). For sdAb‐TIR2, protein levels of PyrF, PyrG, and Cmk were decreased to 7%, 42%, and 11%, respectively, compared to the uninduced control strain upon induction of the respective target sgRNA (Figures S2b). This implies that gene silencing was efficient in the *pyrF* and *cmk* strains, but not in the *pyrG* strain. The relatively high levels of *pyrG* expression seen in the induced strains indicate that the bimodally distributed population in Figure S1e could consist of a growth‐stalled, high‐producing population with little to no expression of *pyrG*, and a regular population that has escaped *pyrG* repression and produces normal levels of PyrG and sdAb. This might, for example, depend on an inefficient sgRNA design, and/or on that the high metabolic burden of high‐producing cells creates a strong selection pressure allowing “CRISPR escapers” to take over the population.

sdAb production was significantly in four of the growth decoupling strains compared to the CRISPRi‐induced control strain (*p* = .023, and *p* = .003 for *pyrF* and *cmk* in psdAb‐TIR1, respectively; *p* = .01, and *p* < .001 for *pyrF* and *pyrG* in psdAb‐TIR2, respectively; Figure 2c,d Panel 2). The best‐performing target for psdAb‐TIR2 was *pyrG* with a 2.7‐fold increase in sdAb per OD and an sdAb content of 6.3% of the total protein content, compared to 2.4% in the induced control strain (Figure 2d, Panel 2). For psdAb‐TIR1, *cmk* had a 2.3‐fold increase in sdAb per OD, and a final content of 4.8% sdAb compared to 2.1% in the control strain.

It is not completely clear why different sgRNAs worked better for psdAb‐TIR1 and psdAb‐TIR2. Inhibition of pyrimidine biosynthesis will lead to alterations of the UTP and CTP pools, and these fluctuations will be different depending on whether *pyrF, pyrG*, or *cmk* is inhibited (see results in Section 3.4). Increases and decreases of the UTP and CTP pools can affect expression of the gene encoding the protein to be produced, especially if the nucleotide sequence upstream the gene contains T and C residues. Thus, the efficiency of the *pyrF, pyrG*, and *cmk* targets may vary as the six nucleotides upstream the start codon are different for sdAb‐TIR1 and sAb‐TIR2 (TGGTAA and GAATAT for sdAb‐TIR1 and sAb‐TIR2, respectively). This is worth considering when using nucleotide biosynthesis inhibition to increase production of proteins.

### 3.3 Proteomics analysis of sdAb‐producing growth decoupling strains

Samples taken at the 24‐hr time point were used for proteomics analysis of all strains. One OD unit of each culture was harvested and analyzed as described in Section 2. The resulting proteome dataset was subjected to differential expression analysis and further analyzed to identify enriched GO terms. The dataset can be found in File S2.

### 3.4 Pyrimidine pathway expression

Up‐ and downregulation of genes in the pyrimidine biosynthesis pathway was determined by comparing the sdAb‐producing CRISPRi‐induced strains harboring an sgRNA plasmid (i.e., growth decoupling strains) to the sdAb‐producing CRISPRi‐induced strain with the empty control sgRNA plasmid (i.e., control strains; Table S3). The analysis showed that protein levels were differently regulated depending on the specific sgRNA target. Generally, gene expression in the upper part of the pyrimidine biosynthesis pathway was upregulated upon inhibition of *pyrF*, downregulated upon inhibition of *cmk*, and up‐ or downregulated upon inhibition of *pyrG* (Figure 3). Pyrimidine biosynthesis is known to be controlled by the nucleotide pool through sensing of intracellular levels of UTP and CTP (Turnbough & Switzer, 2008). These pools are expected to vary depending on the specific sgRNA target. In *pyrF*‐inhibited strains, both UTP and CTP pools are expected to decrease as PyrF is operating upstream of UTP and CTP synthesis (Figure 1a). Inhibition of *pyrG* and *cmk* should, on the other hand, result in reduced CTP levels and an increase (or maintenance) of the UTP pool (Fricke, Neuhard, Kelln, & Pedersen, 1995), as these genes encode enzymes responsible for converting UTP to CTP (*pyrG*), or are active in the salvage pathway of pyrimidine synthesis (*cmk*; Figure 1a).

Expression of *pyrBI* and *pyrE* is transcriptionally regulated by intracellular UTP levels through transcription pausing and attenuation (Turnbough & Switzer, 2008; Turnbough, Hicks, & Donahue, 1983). When UTP concentrations are high, the UTP‐rich transcription pause sites are rapidly transcribed by RNA polymerase, allowing the *pyrBI* and *pyrE* attenuators to form translation‐terminating attenuation loops. *pyrBI* is further controlled by reiterative transcription, resulting in an even higher degree of repression and derepression by the UTP pool (Roland, Liu, & Turnbough, 1988). As a result, *pyrBI* and *pyrE* expression is upregulated at low concentrations of UTP, and vice versa (Turnbough & Switzer, 2008). Analysis of the proteomics data showed a significant upregulation (>twofold) of both PyrBI and PyrE upon inhibition of *pyrF*, in agreement with the expected decrease in the UTP pool in *pyrF* strains. In the *pyrG* strains, PyrBI was downregulated and PyrE was unaffected, while both proteins were downregulated in the *cmk* strains. As UTP levels have been shown to increase in a *cmk* mutant strain (Fricke et al., 1995), it is expected that the expression of *pyrBI* and *pyrE* decrease when *cmk* is inhibited. The downregulated PyrBI and maintained PyrE levels in the *pyrG* strains indicates that blocking this gene may lead to an increase in UTP, but not enough to enhance *pyrE* expression. PyrBI was generally more strongly induced and repressed compared to PyrE, most likely due to the extra level of regulation that the *pyrBI* operon is under (Roland et al., 1988). Expression of *pyrF* has also been shown to increase at low UTP levels, and is likely regulated by UTP‐sensitive reiterative transcription (Liu, Heath, & Turnbough, 1994).

*pyrC* and *pyrD* are transcriptionally and translationally regulated by the intracellular CTP pool (Turnbough & Switzer, 2008). When the intracellular ratio of GTP/CTP is low, the initiating transcript nucleotide of *pyrC* and *pyrD* is shifted to a CTP and the messenger RNAs (mRNAs) will form a hairpin loop that prevents the ribosome from binding and translating the genes (Sørensen, Baker, Kelln, & Neuhard, 1993; Wilson, Archer, Liu, & Turnbough, 1992). PyrC and PyrD levels were upregulated more than twofold in the *pyrF* strains, and between 1.3‐1.8‐fold in the *pyrG* strains. Unexpectedly, both PyrC and PyrD were slightly downregulated in the *cmk* strains. This may indicate that inhibition of *cmk* has less impact on CTP levels compared to inhibition of *pyrG*, or that other regulation factors such as PurR‐based repression or GTP pool‐dependent regulation of *pyrC* and *pyrD* is activate during *pyrG* but not *cmk* repression (Jensen, 1989).

Expression of PyrG was upregulated in the *pyrF* strains, while no effect on expression could be seen in the *cmk* strains. Regulation of *pyrG* has not been fully elucidated in *E. coli*; however, the gene seems to be regulated by the CTP pool through start‐site switching similar to *pyrC* and *pyrD* (Turnbough & Switzer, 2008).

Cmk levels were increased in the *pyrF* and *pyrG*‐inhibited strains, which could indicate that the expression of the gene is affected by UTP and CTP levels. Not much is known about the transcriptional regulation of *cmk*, except that it is cotranscribed with ribosomal protein S1, which is transcriptionally repressed by its own protein product (Jensen, Dandanell, Hove‐Jensen, & Willemoës, 2013; Skouv, Schnier, Rasmussen, Subramanian, & Pedersen, 1990).

Overall, the specific regulation pattern seen in the pyrimidine biosynthesis pathway is consistent with existing literature, and in combination with the significantly reduced expression levels of PyrF, PyrG, and Cmk in their respective target strains (Figures 3 and S2), it shows that the sgRNA and dCas9 are efficiently inhibiting expression of their specific gene target.

It is worth noting that a comparison of induced and uninduced dCas9 expression in cells harboring the control sgRNA plasmid revealed that the expression of dCas9 and sdAb did not significantly affect protein levels in the pyrimidine pathway. The only significant exception was *pyrE* (*p* = .0015), which was slightly downregulated in the control strain harboring psdAb‐TIR2 (Table S3). A previous study did also not report differential expression of the pyrimidine pathway in CRISPRi‐expressing strains with no sgRNA target sequence (Cho et al., 2018).

### 3.5 GO enrichment analysis

**Figure 4:**
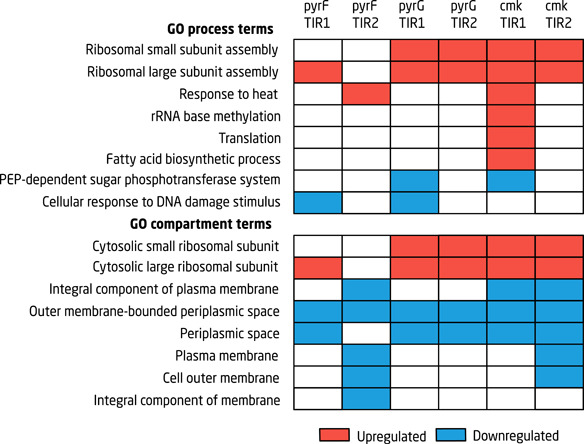
Gene Ontology enrichment analysis. Gene ontology process and compartment terms found to be significantly upregulated (red, *p* < .05) or downregulated (blue, *p* < .05) in the *pyrF, pyrG*, and *cmk* growth decoupling strains expressing sdAB‐TIR1 or sdAb‐TIR2. The differential expression and GO enrichment analysis can be found in File S1. PEP, phosphoenolpyruvate; sdAb, single‐domain antibody [Color figure can be viewed at wileyonlinelibrary.com]

Comparison of sdAb‐producing CRISPRi‐induced strains harboring an sgRNA plasmid (i.e., growth decoupling strains) to the sdAb‐producing CRISPRi‐induced strain with the empty control sgRNA plasmid (i.e., control strains) revealed that several GO process and compartment terms were significantly up‐ or downregulated in the growth decoupling strains (File S2). In total, 1,739 proteins were detected in all samples. Of those, 639, 624, 824, 858, 827, and 516 proteins were differentially expressed in *pyrF* sdAb‐TIR1, *pyrF* sdAb‐TIR2, *pyrG* sdAb ‐TIR1, *pyrG* sdAb—TIR2, *cmk* sdAb—TIR1, and *cmk* sdAb—TIR2, respectively, compared to the control strain (*q* < 0.05). Interestingly, ribosome‐associated terms such as ribosomal assembly and cytosolic ribosomal subunit were upregulated in all growth decoupling strains except *pyrF* harboring psdAb‐TIR2 (Figure 4). It is well known that ribosome content is closely correlated with growth rate in *E. coli* (Miura, Krueger, Itoh, de Boer, & Nomura, 1981). As cells reach the stationary phase, ribosome content decreases drastically and the protein synthesis rate is reduced to around 20% of the rate during exponential growth (Reeve, Amy, & Matin, 1984). The GO enrichment analysis indicates that while the control strains have reached stationary phase after 24 hr, the growth decoupled strains do not enter the stationary phase upon growth inhibition, even though they are no longer growing exponentially. This hypothesis is further corroborated by the relatively low levels of RpoS or σ^38^ found in the growth decoupling strains. *rpoS* expression is normally activated in postexponential and stationary phase in response to a number of inputs, including high cell density, energy limitation, starvation of carbon, and nutrients and changes in osmolarity and pH (Hengge, 2011). In the CRISPRi‐induced *pyrF, pyrG*, and *cmk* strains, RpoS levels were only 7–15% compared to the control strains (Table S4). Several proteins known to be under control of RpoS were significantly downregulated compared to the stationary phase control, including for example pyruvate oxidase, peroxiredoxin, and DNA‐protecting starvation protein (Weber, Polen, Heuveling, Wendisch, & Hengge, 2005) (Table S4). As inhibition of growth decrease the amount of catalytic biomass, the overall glucose uptake rate will decrease even if the specific glucose rate is maintained. This will delay glucose depletion and starvation response, which could explain why no stationary phase response is seen in the *pyrF, pyrG*, and *cmk* strains.

The relatively increased ribosome content may provide an explanation for the increase in sdAb production seen in the growth decoupling strains. With a maintained ribosome availability, growth‐inhibited cells maintain their capacity for protein synthesis during an extended amount of time compared to strains entering stationary phase. Upon CRISPRi‐based inhibition, the cells continue to produce proteins, but cannot divide due to limited nucleotide availability. This hypothesis is corroborated by a previous study, where we used single cell microfluidics to show that GFP is continuously produced in *pyrF*‐inhibited cells (S. Li, Jendresen, Grünberger et al., 2016). Furthermore, as growth is inhibited, glucose cannot be used for biomass accumulation during this time, but is available for other metabolic processes.

Nearly all GO terms that were decreased in the CRISPRi‐induced *pyrF, pyrG*, and *cmk* strains are associated with cellular membrane and periplasmic compartments (Figure 4). There may be several reasons for this. First of all, around 14% of RpoS‐regulated genes encode membrane proteins (Hengge, 2011). Downregulated proteins under control of RpoS include for example transport proteins PotF and UgpB of the ABC superfamily, and putative transport‐ and membrane proteins YdcS and YeaY (Weber et al., 2005; Table S4). Expression of membrane‐associated proteins may also be directly altered by the decrease in pyrimidine supply, as these nucleotides are required for phospholipid synthesis. It has previously been shown that a *cmk* mutant strain with decreased dCTP and CTP pools becomes cold sensitive and displays altered expression of outer membrane porins *ompC* and *ompF* (Fricke et al., 1995). Furthermore, the overexpression of dCas9 can be toxic and alter gene expression in *E. coli* (Cho et al., 2018), which could affect the GO enrichment analysis. A recent study found that expression of dCas9 leads to significant downregulation of cell and membrane biogenesis and translation, and an upregulation of transcription and amino acid and carbohydrate metabolism (Cho et al., 2018). However, a comparison of the CRISPRi‐induced and noninduced control strains in our study did not yield any enriched GO terms (File S2), and we could not identify any overlap in significantly up‐ or downregulated genes and proteins between our data and the dataset from Cho et al. (2018). Our GO enrichment analysis was based on a comparison of growth decoupling and control strains that both express dCas9, which should normalize for effects occurring due to dCas9 expression. Nevertheless, there is still a possibility of unexpected dCas9 effects when there is no sgRNA present to guide DNA binding (Zhang & Voigt, 2018).

It should be noted that membrane and periplasmic proteins are not always reliably quantified with the proteomics method used in this study. However, the fact that membrane protein expression is normally upregulated in stationary phase cells, and that levels of measured membrane proteins is similar within each replicate (Table S4), implies that there is a real decrease when comparing the growth decoupling and the control strains.

Inhibition of *pyrG* was not as efficient as that of *pyrF* or *cmk*. Considering the limited decrease of PyrG protein in the cells, and the fact that GFP distribution in this strain was clearly bimodal (Figure S1e), the proteomics analysis may not give the full picture of up‐ and downregulation of proteins that would occur from complete inhibition of *pyrG* expression. However, the GO terms that were identified overlapped with the ones identified for the *pyrF* and *cmk* strains, and we, therefore, do not consider the limited *pyrG* inhibition to be crucial for the outcome of the proteomics analysis.

### 3.6 Polar effects

Expression of dCas9 and sgRNAs may result in unspecific binding and off‐target effects. Polar effects due to CRISPRi inhibition of *pyrF* resulted in significant downregulation of YciH protein levels (Table S5). Knock‐out of *yciH* has previously been shown to increase expression of 66 genes, and decrease expression of 20 genes in *E. coli* (Osterman et al., 2015); however, none of these proteins were found to be significantly up‐ or downregulated in our study. Polar effects of *pyrG* inhibition resulted in the downregulation of *eno*, located downstream of *pyrG* (Table S5). *Eno* encodes enolase, which catalyzes conversion of 2‐phosphoglycerate to phosphoenolpyruvate, and is also involved in the processing of RNA and degradation of mRNA (Nurmohamed, Adam, Robinson, & Luisi, 2010). It is difficult to predict exactly how these polar effects might have impacted the results of this study. However, in our previous library screen, repression of *eno* and *yciH* did not result in significant inhibition of growth or an increase in GFP production, indicating that the results seen for *pyrF* and *pyrG* inhibition are not affected by downstream *eno* and *yciH* inhibition, respectively (S. Li et al., 2020).

### 3.7 Scale‐up of sdAb production to shake flask fermentation

We applied the best‐performing targets *cmk* for psdAb‐TIR1 and *pyrG* for psdAb‐TIR2 for scale‐up of sdAb production to small‐batch fermentation in shake flasks. The experiment was carried out, as shown in Figure 2a, but cultures were grown in 250‐ml shake flasks with 50 ml media. OD was measured continuously during the experiment (Figure S3a). sdAb expression was induced in all cultures at an OD of 0.4, and samples for proteomics were taken after 24 hr.

**Figure 5:**
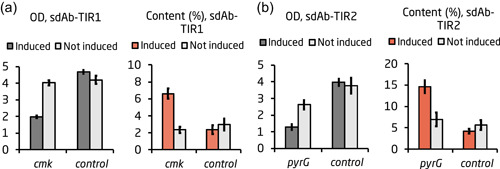
sdAb production in small‐batch fermentation. (a) Growth and sdAb production after 24 hr for strains harboring psdAb‐TIR1 and the *cmk* sgRNA plasmid. (b) Growth and sdAb production after 24 hr for strains harboring psdAb‐TIR2 and the *pyrG* sgRNA plasmid. Cultures, where the CRISPRi system was induced, are shown in the dark gray (OD), red (sdAb content). Uninduced cultures are shown in bright gray. The values were calculated as an average of three biological replicates. Error bars represent standard deviation of the replicates. CRISPRi, CRISPR interference; OD, optical density; sdAb, single‐domain antibody [Color figure can be viewed at wileyonlinelibrary.com]

The replicates with induced CRISPRi expression showed significant growth inhibition with a final OD around half of that in the control strains (Figure 5a,b, Panel 1). Protein levels of Cmk and PyrG in the strains with sgRNAs targeting *pyrG* and *cmk* were decreased to 2.7% and 18%, respectively, compared to the respective induced control strains (Figure S3b). sdAb production was significantly improved in the growth‐inhibited strains (*p* < .001 for both *cmk* and *pyrG* compared to the induced control), with a 2.2‐ and 2.6‐fold increase in sdAb per OD in *cmk* and *pyrG*, respectively (Figure 5b, Panel 2). The final sdAb content reached 6.6% in the *cmk* strain harboring psdAb‐TIR1, and 14.6% in the *pyrG* strain harboring psdAb‐TIR2 (Figure 5b, Panel 2).

## 4 CONCLUSIONS

The nucleotide biosynthesis pathway is an unexpected CRISPRi target for improving protein production, as it provides precursor nucleotides for RNA synthesis. However, inhibiting the essential de novo pyrimidine biosynthesis after initial biomass accumulation still enables cells to supply nucleotides from turnover of RNA and DNA through the pyrimidine salvage pathway, and inhibition of the pyrimidine salvage pathway enables the supply of nucleotides from the de novo pyrimidine biosynthesis pathway. CRISPRi‐based inhibition of *pyrF* and *thyA* has previously been shown to inhibit growth and improve the production of both GFP and mevalonate (S. Li, Jendresen, Grünberger et al., 2016), and in that study, it was further shown that growth decoupled strains remain growth inhibited and metabolically active for up to 48 hr.

In this study, we showed that sdAb production per OD can be increased up to 2.6‐fold upon CRISPRi‐based inhibition of nucleotide biosynthesis. This means that the overall titer of sdAb in shake flask fermentation of the best‐performing growth decoupling strain *cmk* was almost doubled, even though the OD was half of that in the control. The maintained high capacity for protein synthesis and lack of stationary phase response shows that inhibition of nucleotide biosynthesis is a useful approach to increase protein production. Besides the maintained ribosome availability, there are probably other underlying mechanisms in play that enable the growth decoupling strains to maintain or increase production although growth is inhibited. It is possible that the nucleotides supplied from RNA turnover are sufficient to support continued protein synthesis, but not to support DNA replication and cell growth. It would be highly interesting to elucidate the metabolic adjustments upstream of protein synthesis, such as changes in metabolic flux through glycolysis and other pathways, using ^13^C metabolic flux analysis and metabolomics. The occurrence of complete growth and production decoupling could further be confirmed by ribosomal activity assays of cells in the growth‐inhibited state (G. W. Li, Burkhardt, Gross, & Weissman, 2014).

Future efforts should also focus on generating strains that are more industrially applicable, where growth decoupling can be achieved in an autoinducible manner without the use of CRISPRi. This can for example be done by controlling expression of *pyrF, pyrG*, and *cmk* with promoters that automatically turn off when the desired cell density has been reached.

## CONFLICT OF INTERESTS

Jenny Landberg and Alex Toftgaard Nielsen own shares in Mycropt ApS and have a patent on decoupling growth and production by regulation of nucleotide biosynthesis. Naia Risager Wright was employed by the company Novo Nordisk A/S. The other authors declare that there are no conflict of interests.

## AUTHOR CONTRIBUTIONS

J. L. and A. T. N. designed the study. J. L. performed the experimental work and wrote the manuscript. N. R. W. analyzed the proteomics data. T. W. conducted the proteomics analysis. M. H. and A. T. N. supervised the study. All authors read and approved the final manuscript.

## Supporting information

Supporting information

Supporting information

Supporting information

Supporting information

## Data Availability

The data that support the findings of this study are available in the Supporting Information Material of this article.
