# Supplementary material for: CRISPR interference of nucleotide biosynthesis improves production of a single‐domain antibody in Escherichia coli
Source: Biotechnol Bioeng. 2020 Aug 29;117(12):3835–48. doi: 10.1002/bit.27536 (PMC7818426; doi:10.1002/bit.27536)
Supplement: Supplementary file 4 — Supporting information [file BIT-117-3835-s004.pdf]

Supporting information

CRISPR interference of genes involved in nucleotide biosynthesis improves production of a single-domain antibody

Jenny Landberg[1], Naia Risager Wright[1], Tune Wulff[1], Markus J. Herrgård[1,2], Alex Toftgaard Nielsen[1]*

[1] The Novo Nordisk Foundation Center for Biosustainability, Technical University of Denmark, 2800 Kongens Lyngby, Denmark

[2] Current address: BioInnovation Institute, Ole Maaløes Vej 3, 2200 Copenhagen, Denmark

Grants: Novo Nordisk Foundation Grant no. NNF16CC0020908, Novo Nordisk Foundation Grant no. NNF10CC1016517, Innovation Fund Denmark case no. 7038-00165B, Novo Nordisk R&D STAR Fellowship Programme

***Corresponding Author**

Alex Toftgaard Nielsen

The Novo Nordisk Foundation Center for Biosustainability

Technical University of Denmark

Kemitorvet, 2800 Kongens Lyngby

Denmark

Email: atn@biosustain.dtu.dk

Supplementary Table 1

Table S1. Primers used in the study.

Nr.	Primer sequence 5'- 3'	Description
jl37	**TTTACCTTCGTCACCCCATT**GTTTTAGAGCTAG AAATAGCAAGTTAAAATAAGGC	Forward insert of **purA sgRNA** into pSLQ1236
jl38	**AATGGGGTGACGAAGGTAAA**ACTAGTCTTTTCT CTATCACTGATAGGGA	Reverse insert of **purA sgRNA** into pSLQ1236
jl39	**TCCATCGACAGGGGAAACGG**GTTTTAGAGCTAG AAATAGCAAGTTAAAATAAGGC	Forward insert of **purB sgRNA** into pSLQ1236
jl40	**CCGTTTCCCCTGTCGATGGA**ACTAGTCTTTTCTC TATCACTGATAGGGA	Reverse insert of **purB sgRNA** into pSLQ1236
jl41	**CGGGTTTTCCGTGCTGTATA**GTTTTAGAGCTAG AAATAGCAAGTTAAAATAAGGC	Forward insert of **purC sgRNA** into pSLQ1236
jl42	**TATACAGCACGGAAAACCCG**ACTAGTCTTTTCT CTATCACTGATAGGGA	Reverse insert of **purC sgRNA** into pSLQ1236
jl43	**CGGCGACTGGGCCGCTTTCC**GTTTTAGAGCTAG AAATAGCAAGTTAAAATAAGGC	Forward insert of **purD sgRNA** into pSLQ1236
jl44	**GGAAAGCGGCCCAGTCGCCG**ACTAGTCTTTTCT CTATCACTGATAGGGA	Reverse insert of **purD sgRNA** into pSLQ1236
jl45	**GACACGCGCCGGATTATTGC**GTTTTAGAGCTAG AAATAGCAAGTTAAAATAAGGC	Forward insert of **purE sgRNA** into pSLQ1236
jl46	**GCAATAATCCGGCGCGTGTC**ACTAGTCTTTTCT CTATCACTGATAGGGA	Reverse insert of **purE sgRNA** into pSLQ1236
jl47	**TCATAAATCGACTGGTTAAC**GTTTTAGAGCTAG AAATAGCAAGTTAAAATAAGGC	Forward insert of **purF sgRNA** into pSLQ1236
jl48	**GTTAACCAGTCGATTTATGA**ACTAGTCTTTTCTC TATCACTGATAGGGA	Reverse insert of **purF sgRNA** into pSLQ1236
jl49	**AAACACTGAGCAGAGCGCGG**GTTTTAGAGCTA GAAATAGCAAGTTAAAATAAGGC	Forward insert of **purH sgRNA** into pSLQ1236
jl50	**CCGCGCTCTGCTCAGTGTTT**ACTAGTCTTTTCTC TATCACTGATAGGGA	Reverse insert of **purH sgRNA** into pSLQ1236
jl51	**GGCCTAACTGCCCGTTACCG**GTTTTAGAGCTAG AAATAGCAAGTTAAAATAAGGC	Forward insert of **purK sgRNA** into pSLQ1236

jl52	**CGGTAACGGGCAGTTAGGCC**ACTAGTCTTTTCT CTATCACTGATAGGGA	Reverse insert of **purK sgRNA** into pSLQ1236
jl53	**ATTCGGAATGCCGACAGTGC**GTTTTAGAGCTAG AAATAGCAAGTTAAAATAAGGC	Forward insert of **purL sgRNA** into pSLQ1236
jl54	**GCACTGTCGGCATTCCGAAT**ACTAGTCTTTTCT CTATCACTGATAGGGA	Reverse insert of **purL sgRNA** into pSLQ1236
jl55	**GGCATCTTTGTAGCTAAGAG**GTTTTAGAGCTAG AAATAGCAAGTTAAAATAAGGC	Forward insert of **purM sgRNA** into pSLQ1236
jl56	**CTCTTAGCTACAAAGATGCC**ACTAGTCTTTTCTC TATCACTGATAGGGA	Reverse insert of **purM sgRNA** into pSLQ1236
jl57	**CTGTAAATTACTTCCGTTGC**GTTTTAGAGCTAG AAATAGCAAGTTAAAATAAGGC	Forward insert of **purN sgRNA** into pSLQ1236
jl58	**GCAACGGAAGTAATTTACAG**ACTAGTCTTTTCT CTATCACTGATAGGGA	Reverse insert of **purN sgRNA** into pSLQ1236
jl59	**GAGAACCGAAGTCCAGAATG**GTTTTAGAGCTAG AAATAGCAAGTTAAAATAAGGC	Forward insert of **guaA sgRNA** into pSLQ1236
jl60	**CATTCTGGACTTCGGTTCTC**ACTAGTCTTTTCTC TATCACTGATAGGGA	Reverse insert of **guaA sgRNA** into pSLQ1236
jl61	**CGGTAGAGTGAGCAGGAACG**GTTTTAGAGCTA GAAATAGCAAGTTAAAATAAGGC	Forward insert of **guaB sgRNA** into pSLQ1236
jl62	**CGTTCCTGCTCACTCTACCG**ACTAGTCTTTTCTC TATCACTGATAGGGA	Reverse insert of **guaB sgRNA** into pSLQ1236
jl63	**ATGATATGTTTCTGATATAG**GTTTTAGAGCTAG AAATAGCAAGTTAAAATAAGGC	Forward insert of **pyrB sgRNA** into pSLQ1236
jl64	**CTATATCAGAAACATATCAT**ACTAGTCTTTTCTC TATCACTGATAGGGA	Reverse insert of **pyrB sgRNA** into pSLQ1236
jl65	**GCGGCGGATCTTTAATACCT**GTTTTAGAGCTAG AAATAGCAAGTTAAAATAAGGC	Forward insert of **pyrC sgRNA** into pSLQ1236
jl66	**AGGTATTAAAGATCCGCCGC**ACTAGTCTTTTCT CTATCACTGATAGGGA	Reverse insert of **pyrC sgRNA** into pSLQ1236
jl67	**AAAAGGGCTTTACGAACGAA**GTTTTAGAGCTAG AAATAGCAAGTTAAAATAAGGC	Forward insert of **pyrD sgRNA** into pSLQ1236
jl68	**TTCGTTCGTAAAGCCCTTTT**ACTAGTCTTTTCTC TATCACTGATAGGGA	Reverse insert of **pyrD sgRNA** into pSLQ1236

jl69	**TAAGCGCAAATTCAATAAAC**GTTTTAGAGCTAGAAATAGCAAGTTAAAATAAGGC	Forward insert of **pyrE sgRNA** into pSLQ1236
jl70	**GTTTATTGAATTTGCGCTTA**ACTAGTCTTTTCTCTATCACTGATAGGGA	Reverse insert of **pyrE sgRNA** into pSLQ1236
jl71	**AGGAGAATTCGTAACAGCGC**GTTTTAGAGCTAGAAATAGCAAGTTAAAATAAGGC	Forward insert of **pyrF sgRNA** into pSLQ1236
jl72	**GCGCTGTTACGAATTCTCCT**ACTAGTCTTTTCTCTATCACTGATAGGGA	Reverse insert of **pyrF sgRNA** into pSLQ1236
jl73	**GGCAATGCCTTTACCCAGAG**GTTTTAGAGCTAGAAATAGCAAGTTAAAATAAGGC	Forward insert of **pyrG sgRNA** into pSLQ1236
jl74	**CTCTGGGTAAAGGCATTGCC**ACTAGTCTTTTCTCTATCACTGATAGGGA	Reverse insert of **pyrG sgRNA** into pSLQ1236
jl75	**TTTATAGACGGGTTTTGCAT**GTTTTAGAGCTAGAAATAGCAAGTTAAAATAAGGC	Forward insert of **pyrH sgRNA** into pSLQ1236
jl76	**ATGCAAAACCCGTCTATAAA**ACTAGTCTTTTCTCTATCACTGATAGGGA	Reverse insert of **pyrH sgRNA** into pSLQ1236
jl77	**GGCCATCAATGGTAATAACC**GTTTTAGAGCTAGAAATAGCAAGTTAAAATAAGGC	Forward insert of **cmk sgRNA** into pSLQ1236
jl78	**GGTTATTACCATTGATGGCC**ACTAGTCTTTTCTCTATCACTGATAGGGA	Reverse insert of **cmk sgRNA** into pSLQ1236
jl79	**ACGTTTTTTGCTACCGCGTT**GTTTTAGAGCTAGAAATAGCAAGTTAAAATAAGGC	Forward insert of **ndk sgRNA** into pSLQ1236
jl80	**AACGCGGTAGCAAAAAACGT**ACTAGTCTTTTCTCTATCACTGATAGGGA	Reverse insert of **ndk sgRNA** into pSLQ1236
jl130	ACATGTTC**U**TTCCTGCGTTATCCC	Forward primer for amplifying psdAb-TIR1/2 to change origin of replication to CloDF13
jl131	ATCTTTTC**U**ACGGGGTCTGACGC	Reverse primer for amplifying psdAb-TIR1/2 to change origin of replication to CloDF13
jl154	AGAAAAGA**U**CAAATAGCTAGCTCACTCGG	Forward primer for amplifying pCDF-Duet CloDf13 origin
jl155	AGAACATG**U**AAATCTAGAGCGGTTCAGTAG	Reverse primer for amplifying pCDF-Duet CloDf13 origin

Supplementary Table 2

Table S2. Strains and plasmids used in the study. The name used to refer to a specific strain or plasmid in the main text is written in bold.

Strain	Description	Reference/source
E. coli NEB 5-alpha	*fhuA2 Δ(argF-lacZ)U169 phoA glnV44 Φ80 Δ(lacZ)M15 gyrA96 recA1 relA1 endA1 thi-1 hsdR17*; cloning strain	New England Biolabs
E. coli K12 MG1655	F- lambda- *ilvG- rfb-50 rph-1*	Lab collection
SIJ19	**MG1655-gfp**: MG1655 Bb_J23100 *gfp* ::FRT	(Bonde et al., 2016)
E.coli MG1655 (DE3)	**MG1655-DE3**: MG1655 λ*(DE3)*	(Mundhada et al., 2016)
JL100	**MG1655-DE3-dCas9**: MG1655 λ*(DE3)* attB-186(O): tetR-Ptet-dCas9	This study
JL86	MG1655-gfp with psgRNA-purA and pdCas9	This study
JL87	MG1655-gfp with psgRNA-purB and pdCas9	This study
JL88	MG1655-gfp with psgRNA-purC and pdCas9	This study
JL89	MG1655-gfp with psgRNA-purD and pdCas9	This study
JL90	MG1655-gfp with psgRNA-purE and pdCas9	This study
JL91	MG1655-gfp with psgRNA-purF and pdCas9	This study
JL92	MG1655-gfp with psgRNA-purH and pdCas9	This study
JL93	MG1655-gfp with psgRNA-purK and pdCas9	This study
JL94	MG1655-gfp with psgRNA-purL and pdCas9	This study
JL95	MG1655-gfp with psgRNA-purM and pdCas9	This study
JL96	MG1655-gfp with psgRNA-purN and pdCas9	This study
JL97	MG1655-gfp with psgRNA-guaA and pdCas9	This study
JL98	MG1655-gfp with psgRNA-guaB and pdCas9	This study
JL99	MG1655-gfp with psgRNA-pyrB and pdCas9	This study
JL101	MG1655-gfp with psgRNA-pyrD and pdCas9	This study
JL102	MG1655-gfp with psgRNA-pyrE and pdCas9	This study
JL103	MG1655-gfp with psgRNA-pyrF and pdCas9	This study
JL104	MG1655-gfp with psgRNA-pyrG and pdCas9	This study
JL105	MG1655-gfp with psgRNA-pyrH and pdCas9	This study

JL214	MG1655-DE3-dCas9 with psgRNA-pyrF and psdAb-TIR1	This study
JL215	MG1655-DE3-dCas9 with psgRNA-pyrG and psdAb-TIR1	This study
JL216	MG1655-DE3-dCas9 with psgRNA-cmk and psdAb-TIR1	This study
JL217	MG1655-DE3-dCas9 with psgRNA-blank and psdAb-TIR1	This study
JL218	MG1655-DE3-dCas9 with psgRNA-pyrF and psdAb-TIR2	This study
JL219	MG1655-DE3-dCas9 with psgRNA-pyrG and psdAb-TIR2	This study
JL220	MG1655-DE3-dCas9 with psgRNA-cmk and psdAb-TIR2	This study
JL221	MG1655-DE3-dCas9 with psgRNA-blank and psdAb-TIR2	This study

**Plasmid**	**Description**	**Reference/source**
pCDF-Duet	Cloning and expression vector; SpecR	Novagen
pdCas9-bacteria	**pdCas9**: p15A vector with aTc-inducible expression of dCas9; ChlorR	(Larson et al., 2013)
pSLQ1236	**pSLQ1236**: pColE1 vector with aTc-inducible expression of sgRNA; AmpR	(Larson et al., 2013)
pOSIP KO	Cloning-integration plasmid, integrates at the phage 186 site; KanR	(St-Pierre et al., 2013)
pOSIP KO-dCas9	Plasmid for genomic integration of aTc-inducible dCas9; KanR	(Li et al., 2016)
pE-FLP	Plasmid for expressing FLP recombinase to excise the integration plasmid backbone; AmpR	(St-Pierre et al., 2013)
pET28a-Nanobody®-$TIR_{SynEvo1}$	pBR322/ROP vector with Nanobody® expressed from the T7 promoter. Synthetically evolved translation initiation region (TIR) for optimized expression; KanR	(Rennig et al., 2018)
pET28a-Nanobody®-$TIR_{SynEvo2}$	pBR322/ROP vector with Nanobody® expressed from the T7 promoter. Synthetically evolved TIR for optimized expression; KanR	(Rennig et al., 2018)
psgRNA-blank	**control**: pSLQ1236 without sgRNA sequence; AmpR	(Li et al., 2016)
pJL45	**purA**: pSLQ1236 with sgRNA targeting purA; AmpR	This study
pJL46	**purB**: pSLQ1236 with sgRNA targeting purB, AmpR	This study
pJL51	**purC**: pSLQ1236 with sgRNA targeting purC; AmpR	This study
pJL52	**purD**: pSLQ1236 with sgRNA targeting purD; AmpR	This study
pJL53	**purE**: pSLQ1236 with sgRNA targeting purE; AmpR	This study

pJL54	**purF**: pSLQ1236 with sgRNA targeting purF; Amp^{R}	This study
pJL55	**purH**: pSLQ1236 with sgRNA targeting purH; Amp^{R}	This study
pJL56	**purK**: pSLQ1236 with sgRNA targeting purK; Amp^{R}	This study
pJL57	**purL**: pSLQ1236 with sgRNA targeting purL; Amp^{R}	This study
pJL58	**purM**: pSLQ1236 with sgRNA targeting purM; Amp^{R}	This study
pJL59	**purN**: pSLQ1236 with sgRNA targeting purN; Amp^{R}	This study
pJL60	**guaA**: pSLQ1236 with sgRNA targeting guaA; Amp$^{R;}$	This study
pJL61	**guaB**: pSLQ1236 with sgRNA targeting guaB; Amp^{R}	This study
pJL62	**pyrB**: pSLQ1236 with sgRNA targeting pyrB; Amp^{R}	This study
pJL64	**pyrD**: pSLQ1236 with sgRNA targeting pyrD; Amp^{R}	This study
pJL65	**pyrE**: pSLQ1236 with sgRNA targeting pyrE; Amp^{R}	This study
pJL66	**pyrF**: pSLQ1236 with sgRNA targeting pyrF; Amp^{R}	This study
pJL67	**pyrG**: pSLQ1236 with sgRNA targeting pyrG; Amp^{R}	This study
pJL68	**pyrH**: pSLQ1236 with sgRNA targeting pyrH; Amp^{R}	This study
pJL69	**cmk**: pSLQ1236 with sgRNA targeting cmk; Amp^{R}	This study
pJL70	**ndk**: pSLQ1236 with sgRNA targeting ndk; Amp^{R}	This study
pJL97	**psdAb-TIR1**: pET28a-Nanobody®-TIR$_{SynEvo1}$ plasmid with origin of replication changed to ClodF13; Kan^{R}	This study
pJL98	**psdAb-TIR2**: pET28a-Nanobody®-TIR$_{SynEvo1}$ plasmid with origin of replication changed to ClodF13; Kan^{R}	This study

Supplementary Figure 1

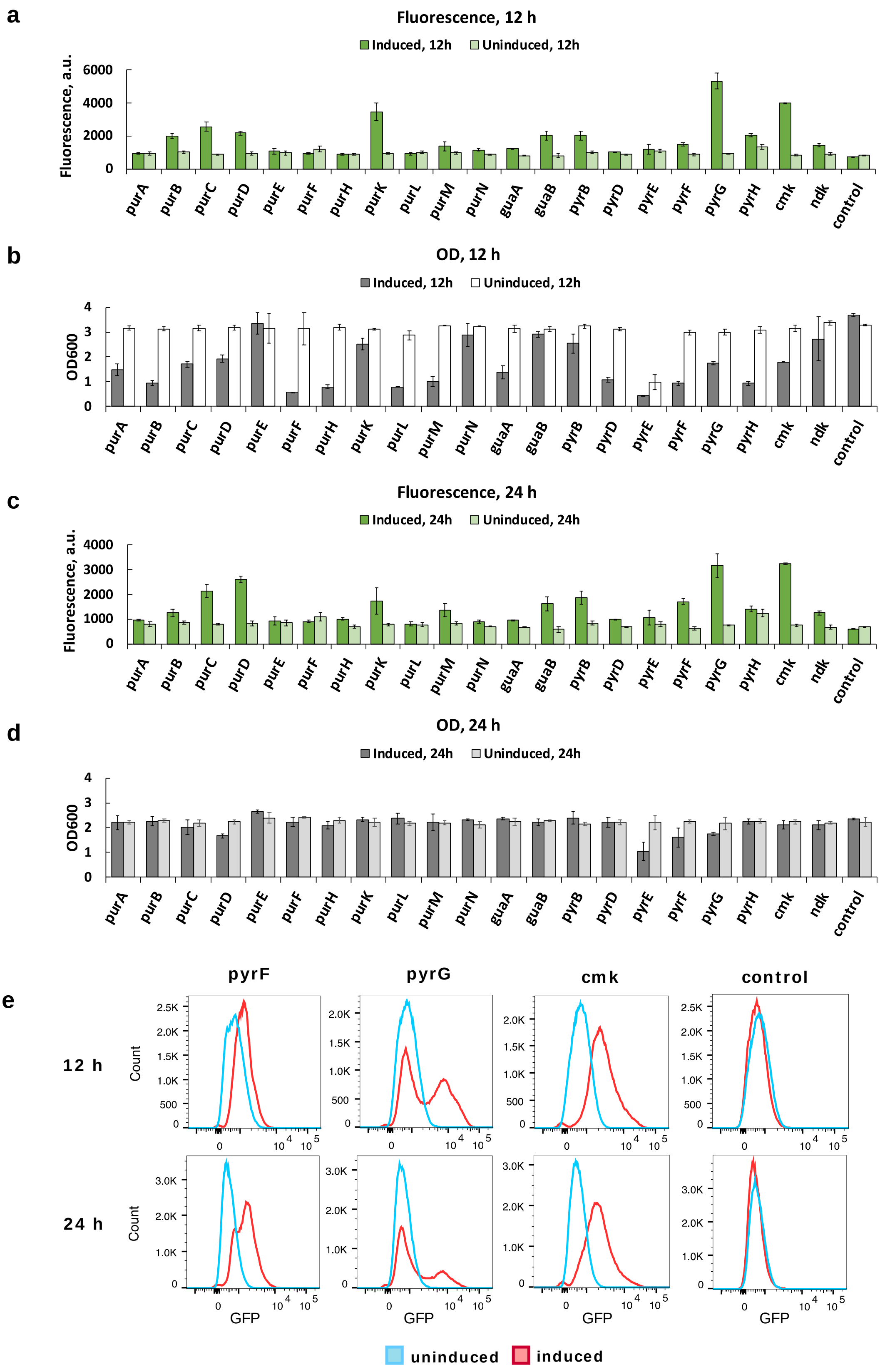

Figure S1. Screening the purine and pyrimidine biosynthesis pathway for growth decoupling targets. (a) GFP fluorescence in CRISPRi-induced and uninduced strains after 12 h of growth. (b) OD of CRISPRi-induced and uninduced strains after 12 h of growth. (c) GFP fluorescence in CRISPRi-induced and uninduced strains after 24 h of growth. (d) OD of CRISPRi-induced and uninduced strains after 24 h of growth. (e) Histograms over 12 h and 24 h GFP fluorescence in CRISPRi-induced and not induced cultures harboring sgRNA plasmids targeting *pyrF, pyrG, cmk* or an empty control vector. The OD and fluorescence were calculated as the average of three biological replicates. Standard deviations between replicates are shown as error bars. CRISPRi-induced strains are shown in dark green (fluorescence in bar plots), dark grey (OD) or red (fluorescence in histograms), and uninduced strains are shown in bright green (fluorescence in bar plots), bright grey (OD) or blue (fluorescence in histograms).

Supplementary Figure 2

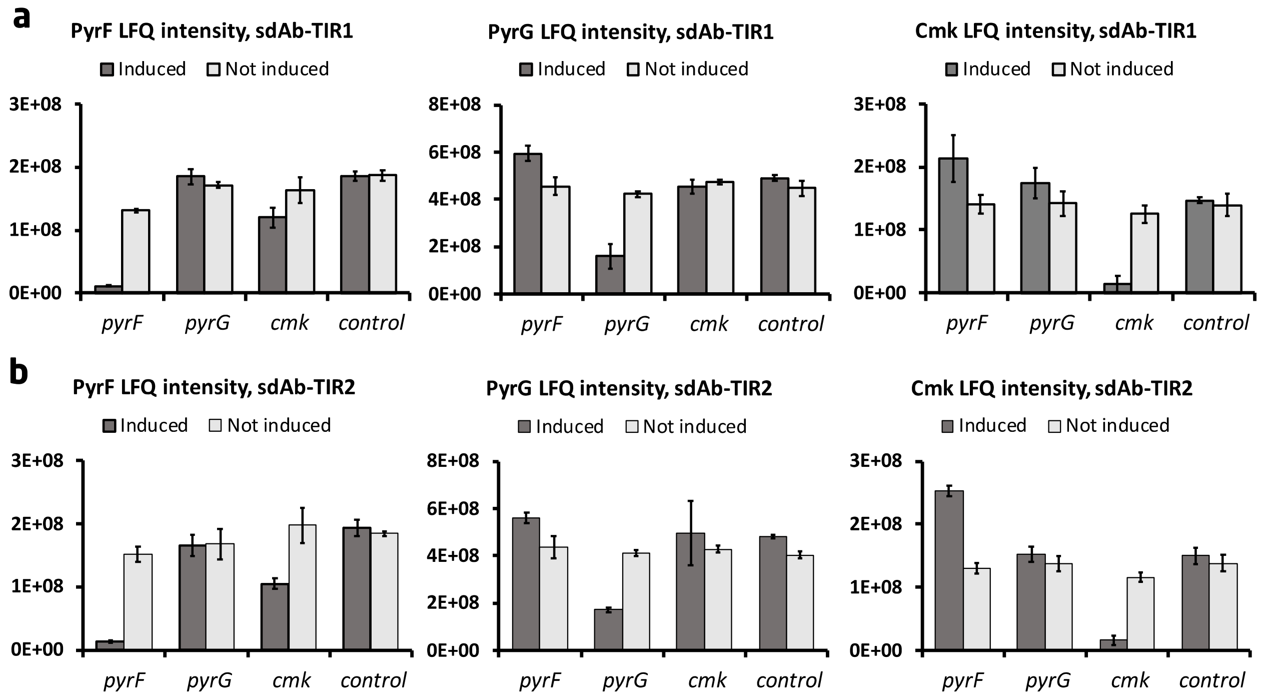

Figure S2. Protein levels of PyrF, PyrG and Cmk in sdAb production strains with or without induction of the CRISPRi growth decoupling system. (a) Protein levels of PyrF (panel 1), PyrG (panel 2) and Cmk (panel 3) in strains harboring the psdAb-TIR1 plasmid and sgRNA plasmids targeting *pyrF, pyrG, cmk* or an empty control vector. (a) Protein levels of PyrF (panel 1), PyrG (panel 2) and Cmk (panel 3) in strains harboring the psdAb-TIR2 plasmid and sgRNA plasmids targeting *pyrF, pyrG, cmk* or an empty control vector. CRISPRi-induced strains are shown in dark grey and uninduced strains are shown in bright grey. Protein LFQ intensities are normalized to OD and were calculated as the average of three biological replicates. Standard deviations between replicates are shown as error bars. The proteome data set can be found in Supplementary File S1.

Supplementary Figure 3

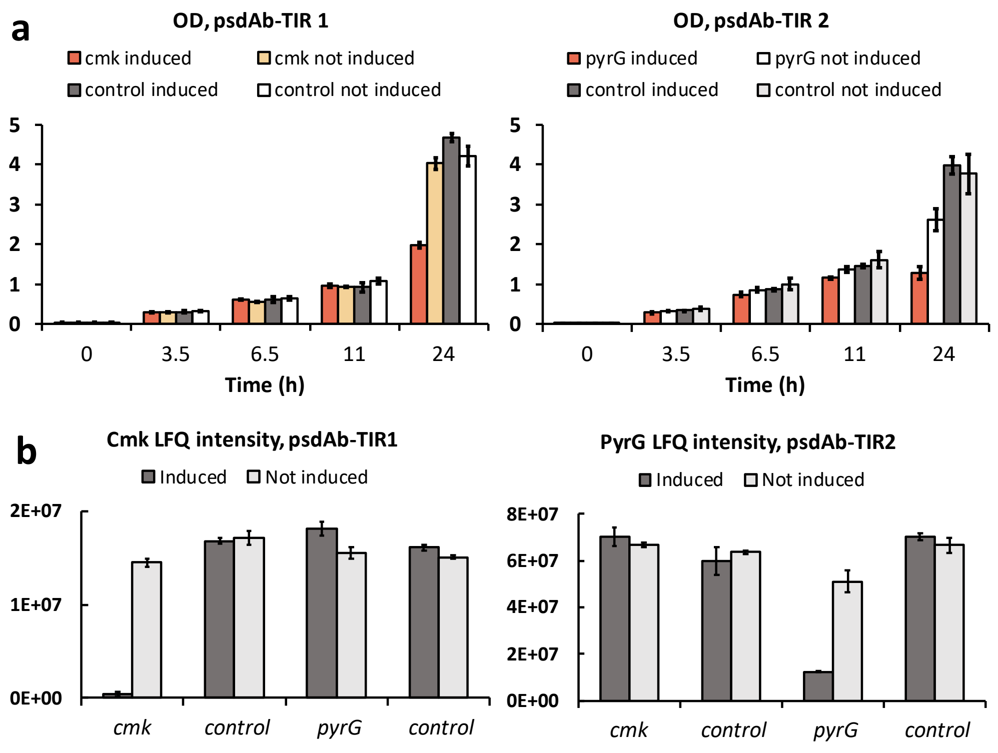

Figure S3. Growth and protein levels during production of sdAb in shake flask fermentation. (a) OD of CRISPRi-induced and not induced strains harboring psdAb-TIR1 and an sgRNA plasmid targeting *cmk* or an empty control vector (panel 1), and psdAb-TIR2 and an sgRNA plasmid targeting *pyrG* or an empty control vector (panel 2). CRISPRi-induced strains are shown in dark orange (*cmk* and *pyrG*) or dark grey (control), and uninduced strains are shown in bright orange (*cmk* and *pyrG*) or bright grey (control). (b) Protein levels of Cmk in strains harboring the psdAb-TIR1 plasmid and sgRNA plasmids targeting *cmk* or an empty control vector (panel 1). Protein levels of PyrG in strains harboring the psdAb-TIR2 plasmid and sgRNA plasmids targeting *pyrG* or an empty control vector (panel 2). CRISPRi-induced strains are shown in dark grey and uninduced strains are shown in bright grey. LFQ intensities are normalized to OD. The OD and protein LFQ intensities were calculated as the average of three biological replicates. Standard deviations between replicates are shown as error bars. The proteome data set can be found in Supplementary File S1.
